# A Rare Case Report of a Tail-Gut Cyst from a Gynecological Point of View 

**Published:** 2018-06

**Authors:** Maroudias George, Gouloumi Alina-Roxani, Tsakiraki Zoi, Oikonomou Maria, Chrelias George, Chrelias Charalampos

**Affiliations:** 1 Department of Obstetrics and Gynecology, Attikon Hospital, School of Medicine, National and Kapodistrian University of Athens, Athens, Greece; 2 Department of Pathology, Attikon University Hospital, School of Medicine, University of Athens, Athens, Greece

**Keywords:** Tail-Gut Cyst, Endometriosis, Dyspareunia

## Abstract

A tail-gut cyst can be often a misleading clinical entity. In half of the patients there is no presenting symptom. On the other half, the patients most commonly present with a variety of symptoms such as rectal pain, constipation, lower back pain, dysuria or dyspareunia. The recommended treatment of choice for the tail-gut cyst is complete surgical excision without rupture of the cyst. We present the case of a 29-year-old female with history of dyspareunia over a 5-month period, who discovered an “ovarian” cyst during an annual scheduled ultrasound appointment. However, the intraoperative findings were surprising. The bottomline is always to keep in mind the Pandora’s Box of the retrorectal space.

## Introduction

With the present case report, we want to shed light in a forgotten and rare diagnosis of tail-gut duplication cyst. Indeed it’s an uncommon congenital cyst found in the retrorectal (presacral) space. According to Mayo clinic data, the estimated incidence of retrorectal lesions is approximately 1 in 40000 (1). There is a strong predisposition to females, with a female to male ratio of 5:1 (1). The tail-gut cyst most commonly presents between ages 30-60, with a mean age of 35 (2, 3). In 1928, Peyron was the first who investigated the embryological tail-gut. In 1938, Gious and Stout introduced the term “tail-gut vestige” in the English literature. In 1961, Edwards proposed the term “retrorectal cyst hamartoma”. In 1987, Hjermstad and Helwig suggested the term “tail-gut cyst”. The latter is a term more descriptive and easily reproducible. The tail-gut cyst almost always occurs in the retrorectal space. From an anatomical point of view this space is a potential space bounded anteriorly from the rectum, posteriorly by the sacrum, inferiorly by the levatorani muscle, superiorly by a peritoneal reflexion and laterally by the ureters (4). The primitive gut is a tube-like structure with a cephalad and caudal blind ends, the foregut and the hindgut respectively. Between them, there is the middle part or midgut (5). During the 4^th^ week of normal embryological development, the embryo begins to fold in a longitudinally and lateral way. Because of this normal folding, the cloacal membrane becomes more ventral comparing to the caudal part of the hindgut and finally encloses it. That’s how the tail-gut is normally formed. The latter structure involutes until the 8^th^ week of development, but if not it becomes a congenital/developmental retrorectal cyst (5). In half of the patients there is no presenting symptom and the tail-gut cyst is incidentally found during routine examination (2). On the other half, the patients most commonly present with a variety of symptoms such as rectal pain, constipation, lower back pain, dysuria or dyspareunia (2). Tail-gut cysts can be complicated by infection, malignant transformation, rupture or fistula formation (5, 6). The first imaging modality which is used for the diagnosis of the tail-gut cyst is transrectal or transvaginal ultrasound. Most commonly, a large cyst with internal echoes is found due to the multi-cystic appearance or the presence of gelatinous material/inflammatory debris within the cyst (7). These misleading findings are very common in other diseases as well, such as endometriosis, teratoma, chordomas, anterior sacral meningioceles or enteric duplication cyst. When there is a diagnostic dilemma, a CT scan should be ordered which is a more sensitive imaging technique (8). A tail-gut cyst is often seen as a discrete, well-marginated mass of the retrorectal space with water or soft tissue density (9). The recommended treatment of choice for the tail-gut cyst is complete surgical excision without rupture of the cyst. The frozen section analysis is usually negative for malignancy. In the final pathology report the characteristic findings are a cyst which is lined with any type of fetal or adult gastrointestinal tract epithelium, an underlined fibroconnective tissue stroma and scattered muscle bundles (10).

## Case report

**Figure 1:**
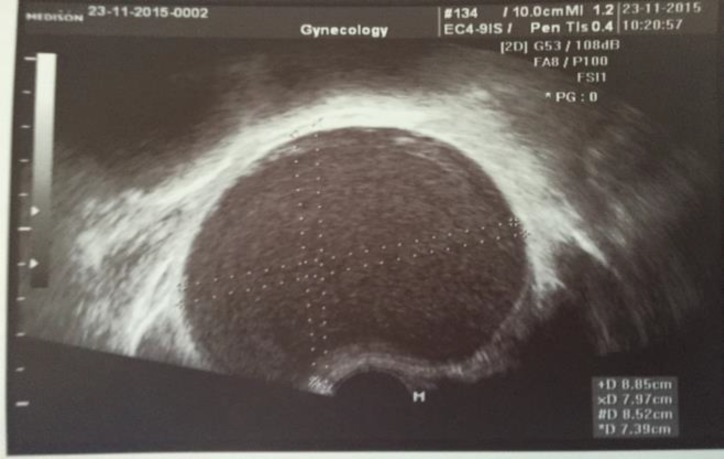
Transvaginal ultrasound, view of the suspicious cyst

We present the case of a 29-year-old female with history of dyspareunia over a 5-month period, who discovered an “ovarian” cyst during an annual scheduled ultrasound appointment. The present study is based on information derived from the patient`s medical record. The study received ethical approval by the Institutional Board of Bioethics of “Attikon" University Hospital (IRB number: 0052/18) and is in accordance with the Declaration of Helsinki and its latter amendments concerning animal and human. According to the ultrasound findings, the patient had a single unicolar cyst (85x74x83mm) which seemed to originate from the right ovary. The cyst had no irregular inner walls or papillary nodules, showed no blood flow during the colored Doppler examination, and inner ground glass appearance (imaging compatible with endometriosis) (Figure 1). 

The physical examination revealed no abdominal sensitivity, however the patient mentioned mild tenderness at cervical examination (chandelier sign positive), especially to the left.

**Figure 2:**
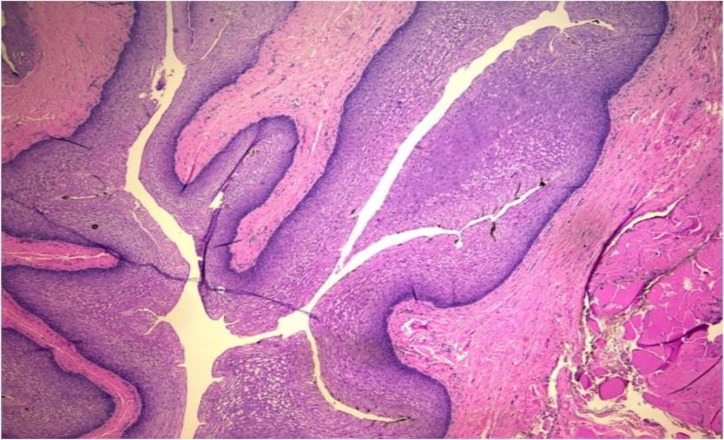
Cyst lined with non-keratinizing squamous epithelium (H&Ex4).

**Figure 3:**
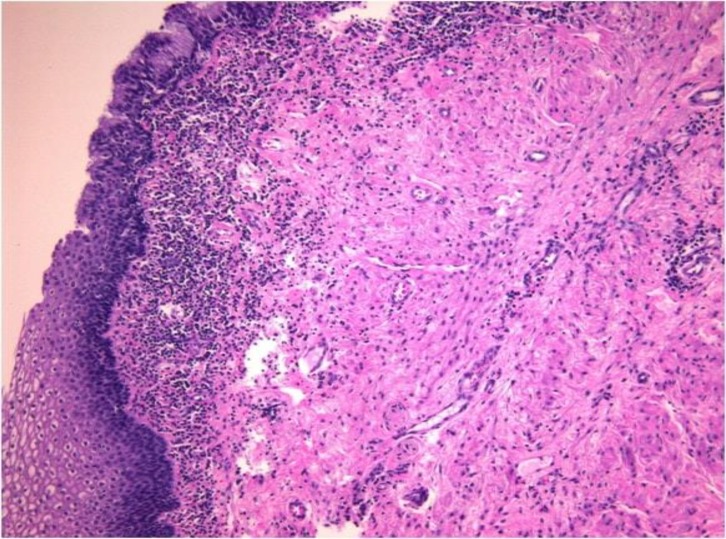
Areas covered by columnar, non-ciliated epithelium containing mucinous cells (H&Ex10)

The anal sphincter had normal tone and the rectal vault was empty while no abnormal masses were palpated. Blood tests revealed white blood cell count and inflammation markers as well as neoplasia markers (AFP, CEA, CA 19/9, CA 125, CA 15/3, b-hCG) within normal rates. However, the intraoperative findings were surprising. The uterus as well as the ovaries had no pathological findings. The patient suffered from a large cyst that originated from the retrorectal space (presacral space), 7x8x9cm in dimensions, which caused symptoms due to its close proximity to the cervix and the anal canal. The cyst was completely resected and sent for histopathological examination. Frozen section analysis was reported as negative for malignancy. Histology disclosed a cystic space lined mostly with non-keratinizing squamous epithelium (Figure 2), with areas covered by columnar, non-ciliated epithelium containing mucinous cells (Figure 3). 

Lack of structured intestinal wall excluded an enterogenous duplication cyst, absence of neural elements a neurenteric duct cyst, whereas absence of other tissues excluded a presacral teratoma (11, 12). No malignancy was documented. Postoperative course was normal and she was discharged after 3 days.

## Discussion

The tail-gut cyst is a rare entity with misleading clinical presentation, which often results in a diagnostic challenge. A history of multiple procedures or drainage attempts should alert the clinician about the possibility of a developmental cyst. In many cases the patients had been misdiagnosed before referral. In the above mentioned case, the patient was referred to our department for abdominal pain, with strong clinical suspicion of endometrioma. Once suspected, the correct diagnosis can be made with a careful and more detailed examination before surgery. The gold standard for the treatment of tail-gut cysts is complete surgical excision. Chronic infection usually complicates 30-50% of the developmental cysts (13). Also due to the risk of malignant degeneration, complete resection with negative margins is recommended (14). So in complicated cases, a multidisciplinary team approach involving gynecologists, colorectal surgeons, neurosurgeons and orthopedists is the safest choice in order to achieve the desirable outcome. For benign cyst, after complete surgical excision, the prognosis is good without the need for adjuvant therapy.

## Conclusion

Which often results in a diagnostic challenge. In complicated cases, a multidisciplinary team approach is the safest choice in order to achieve the desirable outcome.
